# Bridging the evidence-to-action gap: enhancing alignment of national nutrition strategies in Cambodia, Laos, and Vietnam with global and regional recommendations

**DOI:** 10.3389/fnut.2023.1277804

**Published:** 2024-01-08

**Authors:** Tuan Thanh Nguyen, Ngoc Long Huynh, Phuong Nam Huynh, Paul Zambrano, Mellissa Withers, Jennifer Cashin, Sedtha Chin, Roger Mathisen

**Affiliations:** ^1^Alive & Thrive, Global Nutrition, FHI 360, Hanoi, Vietnam; ^2^Keck School of Medicine, University of Southern California, Los Angeles, CA, United States; ^3^Social Marketing & Communication, FHI 360, Washington, DC, United States; ^4^Scientific Management Division, National Institute of Nutrition, Hanoi, Vietnam; ^5^Alive & Thrive, Global Nutrition, FHI 360, Manila, Philippines; ^6^Alive & Thrive, Global Nutrition, FHI 360, Washington, DC, United States; ^7^Alive & Thrive, Global Nutrition, FHI 360, Phnom Penh, Cambodia

**Keywords:** ASEAN, maternal, infant, and young child nutrition (MIYCN), national nutrition strategy (NNS), plan of action for nutrition, Southeast Asia

## Abstract

Nutrition policies are critical frameworks for tackling the triple burden of malnutrition, including undernutrition (i.e., stunting and wasting), overweight, and hidden hunger (i.e., micronutrient deficiencies). We examined (1) the alignment of recent National Nutrition Strategies and Action Plans (NNS) in Cambodia, Laos, and Vietnam with recent global and regional recommendations and standards with a focus on maternal, infant, and young child nutrition and (2) changes compared to the previous NNS. We extracted information regarding the context, objectives, interventions, indicators, strategies, and coordination mechanisms from the most recent NNSs in Cambodia (2019–2023), Laos (2021–2025), and Vietnam (2021–2030). Recent NNSs aimed to reduce malnutrition among priority populations and described program development, monitoring, and evaluation plans for the following interventions: breastfeeding promotion, improved complementary feeding, dietary diversity, safe water, food security, nutritional/health campaigns, strategies for vulnerable groups, and strengthening of policies related to food and nutrition. Direct interventions to improve women’s general nutrition (outside of pregnancy) and adolescent nutrition were not the focus of any NNSs. Although some indicators (e.g., wasting and exclusive breastfeeding) were covered in all recent NNSs, other indicators (e.g., low birth weight and childhood overweight and obesity) were inconsistently incorporated. In comparison to the previous NNS, the following interventions were discontinued in three countries: dietary counseling, maintaining physical activity, monitoring weight gain during pregnancy, maternal micronutrient supplementation, and nutrition and HIV. Despite similarities in structure and content, the recent NNSs of Cambodia, Laos, and Vietnam do not consistently align with global and regional recommendations. Variations in the types of interventions and indicators included may reflect a shift in priorities, attention, or resources. In conclusion, the NNSs of Cambodia, Laos, and Vietnam exhibit both structural and content similarities; however, certain interventions and indicators vary across countries and differ from global and regional recommendations. Enhancing alignment while prioritizing country-specific needs, optimizing coordination, ensuring policy efficacy, and updating nutrition strategy data for cross-country comparisons and knowledge exchange is critical to ensure progress on reducing malnutrition in the region.

## 1 Introduction

Before the COVID-19 pandemic, most countries in the world were already facing a triple burden of malnutrition (1, 2). Globally, nearly 600 million, or 30% of all girls and women aged 15–49 years, are affected by anemia; almost 150 million, or 22% of all children aged 0–5 years, are stunted; and 2.2 billion people are overweight, of whom 772 million are obese (1). Since the COVID-19 outbreak and the war in Ukraine, the number of people facing food insecurity has increased from 148.6 million (in February 2020) to 344.5 million (in June 2022) (3). Food insecurity, both in general and in this specific crisis, has had a significant impact on the nutritional status of the entire population, particularly among those with lower socioeconomic status (3–5). Food insecurity during these crises has led to poor maternal and women’s health outcomes, including increased rates of maternal depression, malnutrition, and death, as well as adverse pregnancy outcomes such as stillbirth and ruptured ectopic pregnancies. Additionally, it has contributed to childhood stunting, wasting, infectious diseases, and mortality (4, 5). To provide an overview of national nutrition policies, plans of action, and programs, the World Health Organization (WHO) conducted a *Global Nutrition Policy Review* in 2009–2010 and 2016–2017 (WHO, 2013, 2018) and, in 2012, adopted six Global Nutrition Targets (GNTs): stunting, anemia, low birth weight, exclusive breastfeeding, wasting, and childhood overweight (1, 2). Countries worldwide are off course on five out of six GNTs (i.e., stunting, anemia, low birth weight, wasting, and childhood overweight), as well as Sustainable Development Goals (SDGs) related to nutrition, including no poverty, zero hunger, good health, and well-being (6), and all diet-related non-communicable disease (NCD) targets (i.e., salt intake, high blood pressure, adult obesity, and diabetes) (1–3). At the current rate of progress, the challenges arising from the war in Ukraine, climate change, and the continued impact of COVID-19 preclude meeting the GNTs and SDGs by 2030 (1, 2).

Most countries in Southeast Asia are experiencing the triple burden of malnutrition, including undernutrition (i.e., stunting and wasting) overweight, and hidden hunger (i.e., micronutrient deficiencies) (7–11). Of the eleven Southeast Asian countries, nine exhibit a high or very high prevalence of stunting (≥ 20%) and wasting (≥ 5%), while five have a medium, high, or very high prevalence of overweight (≥ 5%) among children under 5 years of age (7, 10). Nearly half of children under the age of 5 in Southeast Asia experience micronutrient deficiencies (7, 8, 11). In this region, school-aged children and women also suffer from a high prevalence of malnutrition (7). In a data review of height for people born between 1896 and 1996 in 200 countries, seven of the 11 Southeast Asian countries belong to the lowest 20th percentile for height among adult men and women, and adults in the region showed minimal change in average height from 1896 to 1996 (12). Furthermore, malnutrition in this region is influenced by emerging factors such as inadequate social protection systems, limited access to clean water and sanitation, food insecurity, inadequate dietary quality, the impact of climate change, globalization, urbanization, and evolving agricultural production methods (7, 8, 11).

Comprehensive policies are acknowledged as a pivotal element in a country’s approach to addressing the triple burden of malnutrition (11, 13, 14). Since the inaugural International Conference on Nutrition (ICN) in 1992, nations have adopted national nutrition strategies and action plans (NNS) to eradicate all forms of malnutrition (13, 14). The Association of Southeast Asian Nations (ASEAN) Member States agreed on a regional framework and strategic plan to *End all Forms of Malnutrition* (15). Recently, ASEAN members have endorsed the *Guidelines and Minimum Standards for the Protection, Promotion, and Support of Breastfeeding and Complementary Feeding* (hereinafter referred to as the ASEAN Guidelines) (16), while concurrently developing additional nutrition-related standards for women and children.

Data from the *Report on Nutrition Security in ASEAN* published in 2016 (8) and 2021 (11) shows insufficient progress toward meeting GNTs by 2025, suggesting that in most ASEAN Member States, children start life at a disadvantage, as high rates of stunting, wasting, and overweight are prevalent among children under 5 years of age. Individuals and families encounter a variety of obstacles—economic, physical, social, and cultural—in their pursuit of nutritious diets and access to adequate health and nutrition services, affecting both food environments and the availability of essential, high-quality nutrition services (11). We previously conducted an NNS review of Southeast Asia in 2017, which included Vietnam, Laos, and Cambodia as well as Brunei, Indonesia, Malaysia, Myanmar, the Philippines, and Timor-Leste (17). The review showed that all NNSs included interventions involving antenatal care, micronutrient supplementation during pregnancy, breastfeeding promotion, improved complementary feeding, nutrition in emergencies, and food fortification or dietary diversity. Furthermore, all NNSs had measurable indicators and targets for program monitoring and evaluation plans and addressed collaboration mechanisms, involvement, roles, and responsibilities among stakeholders and sectors. Items found in most, but not all NNSs, included micronutrient supplementation in young children, breastfeeding promotion during pregnancy and support at birth, school feeding, deworming, and treatment of severe acute malnutrition. This review found that despite similarities in the structure and content of Southeast Asian countries’ NNSs, their interventions and indicators varied and did not consistently align with global and regional recommendations. Furthermore, these NNSs did not prioritize issues such as obesity and chronic diseases despite their emergence and burden in Southeast Asia. Some of the gaps identified included a lack of strategies for stakeholder engagement, costing data, and in some NNSs, indicators to track the prevalence of anemia, low birth weight, childhood overweight, breastfeeding, and wasting (17).

All NNSs in the previous review (17) have since been published and updated while the burden of malnutrition has increased. Therefore, a new analysis is needed to examine the progress made toward meeting new targets and aligning national targets with global and regional recommendations. In this paper, we reviewed the contents of the most recent NNS in Cambodia, Laos, and Vietnam; analyzed changes from the previous NNS, and examined their alignment with global and regional recommendations and norms, including the recently released nutrition standards (16) and *Report on Nutrition Security in ASEAN* (11).

### 1.1 Key messages

- National Nutrition Strategies and Action Plans (NNSs) in Cambodia, Laos, and Vietnam adopted a structure and incorporated content that is aligned with the guidelines established during the First International Conference on Nutrition in 1992.
- Recent NNSs are more closely aligned with global and regional recommendations, compared with previous NNSs.
- There is variation across country NNS interventions and indicators, showing inconsistent adherence to global and regional recommendations.

## 2 Subjects and methods

We conducted a desk review of the most recent NNS in Cambodia, Laos, and Vietnam with a focus on maternal, infant, and young child nutrition (MIYCN) (18–20). We extracted information using an extraction form from our previous publication (17), which was developed based on earlier literature (7, 13, 21–26). In addition, we integrated information from the ASEAN Guidelines (16). We heavily adapted the methods used in our previous publication (17) and provide a brief description of the methods as follows.

### 2.1 Policy identification

The full text of the latest NNS documents, as of March 31, 2022 (18–20), was acquired through collaboration with national stakeholders in Cambodia, Laos, and Vietnam. The NNSs were officially translated into English and released by their respective countries. The ASEAN Guidelines were obtained from the ASEAN websites (16). GNTs 2025 (27) and 2030 SDGs (6) were obtained online from the WHO and UN websites. We did not perform similar analyses in other Southeast Asian countries due to a lack of national stakeholders to identify, collect, and translate NNS documents, as well as validate the findings.

### 2.2 Information extraction form

The information extraction form includes information on general characteristics of NNSs, policy context, goals, objectives, strategies, interventions, implementation, monitoring and evaluation, resources, as well as sectors and stakeholders’ roles, policy involvement, and collaboration mechanisms (17). In 2021, WHO and UNICEF released an update to infant and young child feeding (IYCF) practices assessment (28). As a result, new indicators of unhealthy food and beverage consumption were added to this review under the Infant and young child nutrition section of Table 1, (28). Nutrition indicators from ASEAN Guidelines are low birthweight, stunting, wasting, childhood overweight and obesity, iron deficiency anemia, Vitamin A deficiency, exclusive breastfeeding, timely introduction of complementary foods, minimum meal frequency, minimum dietary diversity, and minimum acceptable diet (11). The previous NNS review included nutrition indicators for infants but not for women (29). Due to the high malnutrition burden, this review includes three new nutrition indicators: minimum dietary diversity, minimum meal frequency, and minimum acceptable diet (Table 1) to assess feeding practices for infants and women across ASEAN countries.

**Table 1:** Nutrition indicators included in national nutrition strategies, by country^a^.

	GNTs 2025	SDGs 2030	ASEAN Guidelines	Cambodia		Laos		Vietnam	
			2022	2014–2018	2019–2023	2016–2020	2021–2025	2011–2020	2021–2030
**Infant and young child nutrition**									
Low birthweight	√		√			√	√	√	√
Stunting	√	√	√	√	√	√	√	√	√
Wasting	√	√	√	√	√	√	√		√
Underweight				√	√	√	√	√	
Childhood overweight and obesity	√	√	√		√	√		√	√
Iron deficiency anemia			√	√	√	√	√	√	√
Vitamin A deficiency			√	√				√	√
Iodine deficiency disorders						√		√	√
Early initiation of breastfeeding				√		√			√
Exclusive breastfeeding	√		√	√	√	√	√	√	√
Continued breastfeeding at 1 and 2 years				√					
Timely introduction of complementary foods			√			√			
Minimum meal frequency			√			√			
Minimum dietary diversity			√			√		√	
Minimum acceptable diet Sweet beverage consumption Unhealthy food consumption			√	√	√	√			√
**Nutrition status of women of reproductive age**									
Iron deficiency anemia	√			√	√	√	√	√	√
Chronic energy deficiency (BMI < 18.5 kg/m^2^)				√				√	
Overweight and obesity				√	√			√	√
Minimum dietary diversity									

a GNTs, World Health Assembly’s Global Nutrition Targets; SDGs, United Nations’ Sustainable Development Goals.

### 2.3 Information extraction, management, and analysis

We extracted information from the NNSs and ASEAN Guidelines using an extraction form adapted from our previous study (17). Initially, one researcher reviewed the NNSs and ASEAN Guidelines, and a second researcher conducted a cross-check for accuracy. The findings were then shared with a government representative in Vietnam, staff in Cambodia, and an implementing partner from Save the Children in Laos for verification and input. We provided descriptive findings in tables to allow the comparisons (1) within a country with the older NNS, (2) among other countries, and (3) against regional and global standards. We have also collected information to discuss recent major changes in the NNS of the selected countries and to identify gaps concerning regional and global recommendations.

## 3 Results

Cambodia, Laos, and Vietnam have adopted NNSs as comprehensive national frameworks to improve maternal, infant, and young child nutrition. The most recent NNSs for Cambodia, Laos, and Vietnam were released in 2019, 2021, and 2022, respectively (Table 2). Cambodia and Laos’ NNSs were approved in the same year the policy was effective, while Vietnam’s NNS policy was approved 1 year later (Table 2). The structure of the three NNSs conformed to the framework established by the ICN in 1992 and provided a well-defined description of the country’s context during policy formulation (Supplementary Table S1).

**Table 2:** Characteristics of national nutrition strategies reviewed, by country^a^.

	Date of Approval	Material policy instrument	Governing resources			
			Information, knowledge	Authority	Treasury	Organizational structure
**Cambodia**						
National Strategy for Food Security and Nutrition 2014–2018	Apr. 2014	√	√	√	√	√
National Strategy for Food Security and Nutrition 2019–2023	Nov. 2019	√	√	√	√	√
**Laos**						
National Nutrition Strategy to 2025 and Plan of Action 2016–2020	Dec. 2015	√	√	√	√	√
National Plan of Action on Nutrition 2021–2025	Oct. 2021	√	√	√	√	√
**Vietnam**						
National Nutrition Strategy for 2011–2020, with a vision toward 2030 NPAN Vietnam toward 2015	Feb. 2012	√	√	√	√	√
National Nutrition Strategy for 2021–2030, with a vision toward 2045 and NPAN Vietnam toward 2025	Jan. 2022	√	√	√	√	√

a Categories of policy instrument: material (to result in changes in actual); symbolic (to articulate aspirations for social betterment). Governing resources: information or knowledge (to educate or change behavior of policy targets); authority (to regulate); treasury (to specify the availability and its use of financial resources); organization structure (to stipulate tasks to be done by relevant sectors or stakeholders).

Compared with the previous NNS, the recent NNS included new policy objectives, specifically: (1) to improve diet (Laos), (2) to improve micronutrient status (Cambodia), (3) to prevent and control overweight, obesity, or other chronic diseases (Cambodia), (4) to improve knowledge and practices regarding nutrition in the general population (Laos), (5) to strengthen the national or local health system and workforce (Cambodia), and (6) to reduce inequities or barriers in access to care (Vietnam) (Supplementary Table S1). We listed interventions relating to women at reproductive age, during pregnancy, and the perinatal period (Table 3). Cambodia, Laos, and Vietnam aligned with the regional ASEAN Guidelines on dietary counseling in recent NNSs. Maternal micronutrient supplementation was included in all three countries’ previous and recent NNSs (Table 3). Interventions related to breastfeeding promotion during pregnancy were included in the ASEAN Guidelines and Laos and Vietnam’s NNSs, while Cambodia’s NNS discussed breastfeeding promotion and support but did not specify the timing, e.g., during pregnancy or at birth (Table 3).

**Table 3:** Interventions included in national nutrition strategies, by country.

	Effect^a^	ASEAN Guidelines	Cambodia		Laos		Vietnam	
		2022	2014–2018	2019–2023	2016–2020	2021–2025	2011–2020	2021–2030
**Women at reproductive age during pregnancy and at childbirth**								
Dietary counseling, keeping physically active, or tracking weight gain during pregnancy	3	√	√	√		√	√	√
Balanced energy-protein supplementation for pregnant women	1^b^		√		√		√	
Maternal micronutrient supplementation during pregnancy (including iron folate, calcium, multiple micronutrients, or iodine)	1/ 1^b^		√	√	√	√	√	√
Deworming in pregnancy	1^b^				√		√	
Deliveries supported by skilled attendant	NR							
Breastfeeding promotion during pregnancy (including individual and group counseling)	1	√				√	√	√
Breastfeeding support at birth (including essential newborn care and the Baby-Friendly Hospital Initiative)	1^b^ / 2	√	√		√			
Women’s empowerment, the prevention of domestic violence or gender-based violence	1^b^ / NR	√	√	√	√	√		
Family-planning interventions to promote birth-spacing	2				√			
**Neonates, infants, and young children**								
Breastfeeding promotion (individual and group counseling; including exclusive breastfeeding under 6 months, and prolonged breastfeeding at 1 and 2 years)	1	√	√	√	√	√	√	√
Improved complementary feeding (including timely introduction of complementary foods, dietary diversity, and meal frequency)	1	√	√	√	√	√	√	√
Zinc supplementation (including those for diarrheal children)	1	√	√		√			
Iron supplementation	1^b^	√	√		√	√	√	√
Vitamin A supplementation	1	√	√		√	√	√	√
Deworming	1^b^		√	√	√	√	√	√
Feeding for sick children (including diarrhea and respiratory infection)	NR	√			√		√	
Treatment of moderate / severe acute malnutrition	1	√	√	√	√		√	√
Infectious diseases prevention and management (e.g., diarrhea, acute respiratory infection, malaria)	1/ 1^b^	√		√	√		√	
School feeding programs	3		√	√	√	√	√	√
**Food, food safety and food security**								
Universal salt iodization	1	√	√	√	√		√	√
Food fortification (including Vitamin A, iron)	1^b^	√	√	√	√		√	√
Dietary diversification strategies, small animal husbandry, or home gardening	2	√	√	√	√	√	√	√
Safe water, sanitation, or hygienic practices	1	√	√	√	√	√	√	√
Food safety, quality control, the prevention of food-borne diseases, or food labelling	NR	√	√	√	√		√	√
Food security, food market, and trade	1^b^ /NR	√	√	√	√	√	√	√
Nutrition in emergencies	1^b^	√	√		√	√	√	√
Social safety nets, cash transfers, microcredit programs, food-for-work programs, or generalized food subsidies	1^b^ /NR	√	√	√				
Agricultural production subsidies, land use, or reform	1^b^ /NR		√	√	√		√	
**Nutrition care for disease prevention and treatment**								
Nutrition and HIV	1/ 1^b^	√	√		√		√	
Hypertension, diabetes, or cardiovascular diseases	NR		√		√		√	√
Increased physical activities	NR	√					√	√
Reduction of alcohol consumption or tobacco usage	1					√		
Reduction of sugar and fat-added foods, sweetened beverages, or salt consumption	NR	√		√		√		√
**Cross-cutting strategies**								
Mass communication	3	√	√	√		√	√	√
Interpersonal communication	1	√	√		√		√	√
Nutritional or health campaigns	3	√	√	√	√	√	√	√
Health system strengthening	NR	√	√	√	√	√	√	
Agriculture or food system strengthening	1	√	√	√	√	√	√	√
Specific strategies for vulnerable groups	1/ 1^b^	√	√	√	√	√	√	√
Integrated program monitoring and evaluation	1^b^	√	√	√	√	√	√	
Social or community mobilization	NR	√		√	√	√	√	√
**Supportive policies and legislations**								
Strengthen legislations on food fortification	NR	√	d	√	d	√	d	√
Strengthen legislations on food safety	NR		√		d		√	
Strengthen policies and commitments relating to food and nutrition; or incorporating nutrition goals into relevant laws, regulations, policies, and plans.	NR	√	√	√	√	√	√	√
Strengthen legislations on marketing of breastmilk substitutes	NR	√	√	√	√	√		√
Health insurance to cover nutrition, curative care for young children, or nutrition preventative care	NR	√						√
Strengthen legislations on maternity leave or workplace lactation support	NR	√	√	√			√	√

a Intervention effectiveness on maternal and child nutrition: (1) sufficient evidence; (2) insufficient or variable evidence; and (3) little or no evidence; NR, not reviewed (21, 23, 30).

b Interventions effective in specific context. d, under development.

In comparison with the previous NNS, the following interventions were dropped: dietary counseling, keeping physically active, or tracking weight gain during pregnancy (three countries), maternal micronutrient supplementation (Cambodia, Laos, and Vietnam), deworming during pregnancy (Laos and Vietnam), breastfeeding support at birth (Cambodia and Laos), and family planning (Laos) (Table 3). Balanced energy-protein supplements during pregnancy were dropped in the three countries’ NNSs. Although Cambodia’s NNS briefly shared progress on nutrition-related interventions for women of reproductive age and Laos mentioned the importance of nutrition education for adolescent girls, the three countries’ updated NNSs primarily focused on direct interventions to improve nutrition for young children and women during pregnancy, rather than adolescents and women at reproductive age and in the perinatal period (19, 20).

Regarding interventions for neonates, infants, and young children, improved complementary feeding, school feeding program interventions, and deworming were included in all three NNSs, with the three countries also aligning on regional ASEAN recommendations for improved complementary feeding (Table 3). Interventions dropped in recent NNSs in this category included zinc supplementation in Cambodia and Laos (excluded in both the past and recent NNSs in Vietnam) (Table 3). Iron and Vitamin A supplementation was included in Laos and Vietnam but dropped for Cambodia (Table 3). Treatment of moderate/severe acute malnutrition was mentioned in the NNSs of Cambodia and Vietnam, but not Laos (Table 3). Both Laos and Vietnam dropped infectious disease prevention and management intervention, while this was a new intervention picked up by Cambodia (Table 3). Interventions related nutrition and HIV were dropped in all three countries in the recent versions (Table 3).

Interventions for food, food safety, and food security aligned with the ASEAN Guidelines and were included in all three countries’ recent and previous NNSs (Table 3). For example, these interventions are dietary diversification; safe water, sanitation, or hygienic practices; and food security, food market, and trade (Table 3). Subsidies for agricultural production, land use, or reform were not included as recommended interventions in the ASEAN Guidelines (Table 3). Some interventions were less consistent across all three countries, e.g., food fortification interventions (Cambodia and Vietnam), nutrition in emergencies (Laos and Vietnam), food safety (Cambodia and Vietnam), and social safety nets (Cambodia) (Table 3). Interventions for disease prevention and control were less consistent with ASEAN guidelines and all three countries’ NNSs. Dropped interventions were nutrition and HIV (all three NNSs) and hypertension, diabetes, or cardiovascular diseases (Cambodia and Laos) (Table 3). Newly added interventions not presented in previous NNSs included a reduction of alcohol consumption or tobacco usage (Laos) and a reduction of sugar and fat-added foods, sweetened beverages, or salt consumption (all three countries) (Table 3).

Cross-cutting strategies in all three NNSs included national health campaigns, mass communication, agriculture or food system strengthening, interventions for vulnerable groups, and social or community mobilization (Table 3). Interventions included in some NNSs were interpersonal communication (Vietnam), and health system strengthening (Cambodia and Laos) (Table 3).

Supportive policies and legislation interventions, specifically food fortification regulations and food safety, were in all three NNSs while others, such as health insurance to cover nutrition-related services, were only in Vietnam’s recent NNS and the ASEAN Guidelines. Policies and legislation on the marketing of breastmilk substitutes were included in ASEAN guidelines and the recent NNSs of the three countries (Table 3). Maternity leave and workplace lactation support as a part of maternity protection are only listed in Cambodia and Vietnam’s recent NNSs and ASEAN Guidelines (Table 3). Policy objectives related to non-communicable diseases were included in two NNSs (Cambodia and Vietnam) (Supplementary Table S1). All three NNSs included indicators relating to MIYCN listed in the GNTs. Infant and young child nutrition indicators varied but included: low birth weight (Laos), stunting (all three countries), wasting (all three countries), underweight (Cambodia and Laos), childhood overweight and obesity (Cambodia and Vietnam), iron deficiency anemia (Cambodia and Laos), Vitamin A deficiency (Vietnam), and iodine deficiency disorders (all three countries). While some indicators for breastfeeding practices were in recent NNSs such as early initiation of breastfeeding (Vietnam) and exclusive breastfeeding (all three countries), indicators such as continued breastfeeding at 1 and 2 years were not included (Table 1).

Some nutrition indicators present in previous NNSs were dropped in recent NNSs, resulting in the recent NNSs’ failure to align with regional ASEAN Guidelines. These indicators include the timely introduction of complementary foods (Laos), minimum dietary diversity (Laos and Vietnam), and minimum acceptable diet (Laos).

Recent NNSs include indicators related to the nutrition status of women of reproductive age, including iron deficiency anemia (all three countries) and overweight or obesity (Cambodia and Vietnam) (Table 1). However, chronic energy deficiency (BMI < 18.5 kg/m^2^), which was previously included in Cambodia and Vietnam’s NNSs, was no longer mentioned in any of the three countries’ newest NNSs (Table 1).

As indicated in Table 4, each NNS delineated the roles, responsibilities, collaborative mechanisms, and execution framework, specifying whether the strategy operates within a focal sector, involves multiple sectors, or engages various stakeholders. The roles of governmental stakeholders, both at the national and sub-national levels, were assessed based on their contributions in terms of financial resources, provision of technical support, and implementation (Supplementary Table S2). Typically, the concept of technical support encompassed the involvement of international organizations, donors, the private sector, and academic or research institutions (Supplementary Table S2). Each NNS presented this information uniquely, with variations in the level of detail. Within the content of each NNS, Cambodia, Laos, and Vietnam all dedicated specific sections to elucidate the sectors or stakeholders involved.

**Table 4:** Sectors and stakeholders involved in national nutrition strategies, by country.

	Cambodia		Laos		Vietnam	
	2014–2018	2019–2023	2016–2020	2021–2025	2011–2020	2021–2030
**Sectors involved**						
Health and Nutrition	√	√	√	√	√	√
Agriculture	√	√	√	√	√	√
Food industry	√	√	√			√
Education		√	√	√	√	√
Culture, Information, Communication		√	√	√	√	√
Science, Technology, Environment		√	√	√		√
Labor, Social affairs	√	√	√	√	√	√
Finance		√	√	√		√
Internal and external trade		√	√	√		√
Planning, Investment		√	√	√	√	√
**Stakeholders involved** ^**a**^						
National level (including Government, Parliament, and Ministries)	√	√	√	√	√	√
Sub-national levels (including provincial, city, district, and sub-district local authorities in various sector such as civil, health, nutrition, education agriculture)	√	√	√	√	√	√
Civil society organizations, or unions^b^	√	√	√	√	√	√
International organizations, or donors^c^	√	√	√	√	√	√
Private sector	√	√		√		√
Academic or research institutions	√	√		√	√	√
Contains section dedicated to sector and stakeholder involvement	√	√	√	√	√	√
Clearly describes the roles and responsibilities of sectors and stakeholders	√	√	√	√	√	√
Collaboration mechanism indicated	√	√	√	√	√	√

a Data allow for specific contributions such as technical support, financial support, or implementation are included in Appendix 2.

b Civil society organizations and unions include unions (Trade, Women, Farmers, and Youth), societies (Veterans, Teachers, Elderly), and religious, villages and tribe leaders.

c International Organizations, donors include UNICEF, WHO, FAO, World Bank, other development bank (e.g., ADB), governments of other countries (e.g., USAIDS, Australian Aid, UK Aid), Foundations (e.g., Bill & Melinda Gates Foundation), research foundation, and international non-Governmental Organizations (NGOs), and In-country donors.

## 4 Discussion

### 4.1 Alignment with global and regional standards

For over 25 years, NNSs have played a pivotal role in enhancing nutrition planning, implementation, monitoring, and evaluation, contributing to improved nutrition and health outcomes globally (13, 22). The structures and contents of recent NNSs in Cambodia, Laos, and Vietnam aligned with the 1992 ICN (13), which facilitated information capture and application (13, 20, 22). However, recent NNS are not comprehensive enough to meet global standards (i.e., 2025 GNTs and 2030 SDGs) or ASEAN regional standards despite increased efforts made since the previous NNS review (17). To meet regional standards and address these gaps as nutrition changes in the region from food system globalization, urbanization, and economic growth (11), countries must take steps to standardize process nutrition indicators, promote recommended interventions, and monitor progress toward meeting target indicators (19, 20).

### 4.2 Dynamic changes in the content of NNSs

While the recent NNSs of Laos, Vietnam, and Cambodia include plans to combat malnutrition, they exhibit a notable absence of comprehensive details regarding context, objectives, interventions, indicators, strategies, and coordination mechanisms when compared to their previous NNS documents (20). We compared the most recent NNSs to the previous versions to analyze and explain differences and trends in measuring malnutrition. We identified several noteworthy trends in these three countries. First, some interventions and indicators present in previous NNSs have been omitted in the most recent strategies, including balanced energy-protein supplementation for pregnant women (all three NNSs), zinc supplementation (Cambodia and Vietnam), iron supplementation (Cambodia), universal salt iodization (Laos), and chronic energy deficiency (Vietnam) (Tables 1, 3). Laos’ NNS acknowledged the inclusion of a smaller set of 22 indicators and 36 interventions compared to the previous NNS and attributed the change in indicators from the previous NNS as a response to prioritization in areas such as climate change, gender equality, and nutrition in disasters and emergencies (19, 20). One possible reason was the onset of the COVID-19 pandemic, which emerged in 2020. As a result of the COVID-19 pandemic, Laos’ recent NNS mentions the disruption of nutrition-related services while Cambodia’s “Roadmap for Food Systems for Sustainable Development” highlights the need to strengthen existing systems (e.g., health, economic, agricultural, and food) to better prepare for future events like the COVID-19 pandemic (19, 20). NNS priorities may have shifted due to key decision-making events and meetings held online because of COVID-19 restrictions and may have led to the exclusion of individuals who advocate for and shape countries’ food systems and contribute to plans’ development and adoption. Cambodia’s “Roadmap for Food Systems for Sustainable Development” mentions that the roadmap was developed at the height of the COVID-19 pandemic, and the lack of critical voices and transition to online events occurred because of COVID-19 restrictions (19).

Another potential reason for the reduction in the number of indicators and interventions between previous and recent NNSs is significant progress made on previous indicators and interventions, resulting in a focus on other priorities. For example, iodine was no longer an indicator for Laos’ recent NNS due to improvements in household consumption of iodized salt and children’s iodine levels (18). In Vietnam, there was a 10 years difference since the last NNS release. During this time, it is likely that the NNS’s recent strategy reflected the changing country context regarding a shift in focus to nutrition policies and school nutrition. In Vietnam, chronic energy deficiency (a body mass index of less than 18.5 kg/m^2^) became rare and thus excluded from the recent NNS. Cambodia’s NNS may not fully reflect the change in the landscape due to reliance on outdated data (19) from the most recent nationally representative nutrition survey at the time of publication: the 2014 Cambodia Demographic and Health Survey (CDHS). The most recent CDHS was released after the NNS was released (19). Indicators and targets listed in the three NNS were mostly nutrition indicators. Table 1 includes a list of the six GNTs (27). The only GNTs tracked by all three countries were iron deficiency anemia for women of reproductive age and stunting for children under 5 years. The three countries did not align on the other four GNTs, thus limiting the utility of NNSs to track the region’s contribution and progress toward meeting all six targets by 2025.

Another possible reason for the reduction of indicators in the most recent NNSs is a lack of reliable and available data in areas ranging from interventions and implementation to monitoring and evaluation. For example, Laos revised its nutrition measures and indicators after discovering that the agriculture sector was not able to track these indicators (20). Despite Cambodia NNS shifting focus toward breastfeeding to address the decline in breastfeeding rates over the past few years, there is limited data on the effectiveness and manner in which breastfeeding-related interventions are implemented (19). Data is limited even for areas on which all three countries align, such as dietary diversification strategies, small animal husbandry, or home gardening and safe water, sanitation, or hygienic practices (8).

All three countries’ NNSs lacked information on interventions for women during the preconception period or for adolescents (other than during pregnancy for women at reproductive age), except for Vietnam with the indicator on anemia among 10–14-year-old girls (18–20). This major gap has been highlighted previously in this region and globally, citing a lack of data on the diets of these groups (8, 14, 16, 31). These populations should be a focus for both nutrition and non-nutrition interventions to ensure that women of reproductive age are physically and psychologically ready for pregnancy, thus improving the quality of pregnancy, reducing complications, and improving birth outcomes (7). The lack of data prevents countries from learning, adapting, and applying best practices toward global and regional contexts, and places additional constraints on areas such as decision- and policymaking, coordination, and implementation.

These results underscore the necessity to boost capacity, provide support to governments at various levels to reallocate policy and resources toward evidence-based interventions and programs, and actively implement, monitor, and evaluate multisectoral interventions to address the complex challenge of the triple burden of malnutrition during health emergencies.

These results underscore the need to boost capacity, provide support to governments at various levels to reallocate resources toward evidence-based interventions and programs, and actively implement, monitor, and evaluate multisectoral interventions to tackle the triple burden of malnutrition amid health emergencies (7, 8, 11).

### 4.3 Delays in release date remain a key challenge of NNS

While NNSs are useful in setting goals and measuring national progress on a wide range of nutrition indicators, they also have some limitations. First, for the three countries, there was an approximate one-year delay in the approval compared to the duration of the policy and there was no improvement in terms of the timing compared with the previous policy reviews (17). This means there is at least a one-year gap in the direction and resources for NNS implementation. Evaluating the achievement of a previous NNS and planning for a new one requires progress and impact data from the national survey, surveillance, and monitoring data. Member states should maximize available data from international sources such as UNICEF, WHO, WB, and Global Nutrition Reports to inform the progress. In addition, countries should promote a robust, streamlined, reliable electronic monitoring system for inputs, outputs, outcomes, and impacts to facilitate the planning.

Second, identifying and adopting new interventions or indicators during the drafting or implementation process is challenging. This, combined with the lack of data and insufficient human resource capacity in many ASEAN countries, hinders the effective implementation of nutrition interventions (16). Since countries are unable to use their impact study to inform these interventions, using global and regional evidence can be a good approach to inform interventions and indicators. However, there is also a gap in time between the release of the global guidelines and evidence and the inclusion into NNS. Policymakers would need to wait until the next round of NNS to include new interventions and indicators recommended by global guidelines. The late release of NNS is affected by a lack of evidence, consensus, or champions (32, 33).

Successful planning and implementation of an NNS requires the involvement of different stakeholders and sectors and an understanding of nutrition and health status, priorities, and policies between countries (13, 22). In Cambodia, Laos, and Vietnam, NNSs require further collaboration between sectors and countries involved with addressing malnutrition to ensure cohesion and comparability among NNS frameworks and address the triple burden of malnutrition (11). However, similar to the findings from the Global Nutrition Policy Report (14), we observed that not all NNSs explicitly outlined sector and stakeholder involvement or financial commitments. This omission can hinder governments’ ability to hold stakeholders accountable for their contributions to achieving GNTs. Consequently, the responsibility for nutrition programs tends to fall primarily on the nutrition and health sectors (14), which may prevent the country or region from applying a comprehensive systems approach to promote nutrition and health status effectively, efficiently, and sustainably (7, 14). These study findings may also support ongoing efforts within ASEAN to establish a regional surveillance system among member states.

### 4.4 Study strengths

To the best of our knowledge, we are among the few researchers who have conducted a review of recent NNSs in lower- and middle-income countries, comparing them with recent global and regional recommendations and standards. We evaluated changes compared to previous NNSs. Our study employed standardized methods, including the use of a questionnaire, an information extraction form, and a rigorous review and validation process. These methods have been successfully applied in our previous study, which was peer-reviewed and published.

### 4.5 Study limitations

There are some limitations to our study including only Cambodia, Laos, and Vietnam because of the research team’s access to national stakeholders for identifying, collecting, translating NNS documents, and interpreting and validating the study findings. Nevertheless, our approaches and tools could be applied by other researchers for similar research in different settings. Our findings are primarily focused on comparing national policies with regional and international standards, rather than directly comparing countries. However, the detailed information is available in the data tables.

Finally, we were unable to provide information on intervention implementation or results, as these topics fall beyond the scope of our study. It is important to note that our focus lies on policy analysis rather than evaluating the effectiveness of policies or their implementation. Additionally, we could only provide certain assumptions about the reasons behind the findings, such as the presence or absence of specific indicators or interventions. Further studies would be necessary to delve deeper into these aspects.

## 5 Conclusion

The NNSs of Cambodia, Laos, and Vietnam exhibit structural and content similarities, with a focus on promoting breastfeeding and enhancing complementary feeding. Despite certain alignments, there are some variations among countries and between NNS with international and regional standards. Factors such as COVID-19, shifting priorities, and data availability drove indicator adjustments in NNSs. To enhance coordination and policy efficacy, updating nutrition strategy data for cross-country comparisons and knowledge exchange is vital. Addressing NNS gaps through enhanced capacity, coordination, and governance ensures alignment with regional standards and amplifies the focus on MIYCN. This approach guarantees a successful and sustainable approach across the region.

## Author contributions

TN: Conceptualization, Data curation, Investigation, Methodology, Project administration, Resources, Supervision, Validation, Visualization, Writing – original draft, Writing – review & editing. NH: Conceptualization, Data curation, Formal analysis, Investigation, Methodology, Validation, Visualization, Writing – original draft, Writing – review & editing. PH: Data curation, Validation, Writing – review & editing. PZ: Conceptualization, Data curation, Formal analysis, Investigation, Validation, Writing – review & editing. MW: Conceptualization, Validation, Writing – review & editing. JC: Conceptualization, Validation, Writing – review & editing. SC: Data curation, Validation, Writing – review & editing. RM: Conceptualization, Funding acquisition, Resources, Supervision, Validation, Writing – review & editing.
