# Supplementary material for: Bridging the evidence-to-action gap: enhancing alignment of national nutrition strategies in Cambodia, Laos, and Vietnam with global and regional recommendations
Source: Front Nutr. 2024 Jan 8;10:1277804. doi: 10.3389/fnut.2023.1277804 (PMC10800738; doi:10.3389/fnut.2023.1277804)
Supplement: Supplementary file 1 [file Data_Sheet_1.pdf]

Appendix 1. The context and objectives indicated in the policies

	Cambodia		Laos		Vietnam	
	2014-2018	2019-2023	2016-2020	2021-2025	2011-2020	2021-2030
**Policy context**						
Poverty or economic problems	√	√	√	√	√	√
Issues relating to agriculture, food production, food distribution, food insecurity, or hunger	√	√	√	√	√	√
Population-related factors such as change or an aging population					√	
Health problems of select groups (including mortality or morbidity, low life-expectancy at birth, and HIV)		√		√		√
Poor nutrition status of infants and young children	√	√	√	√		√
Poor nutrition status of adolescents, women, and mothers (including chronic energy malnutrition and short stature (height < 1.45 m))	√	√	√	√	√	√
Micronutriment deficiencies (e.g., Vitamin A, Vitamin D, zinc, iron, iodine)	√	√	√	√	√	√
Nutrition-related chronic diseases (e.g., cardiovascular diseases and risks, including overweight, obesity, hypertension, and diabetes)		√	√	√	√	√
Issues relating to education, gender, women's rights, early marriage, or inequalities		√		√	√	√
Unsafe water or sanitation	√	√	√	√		√
Environment, climate change, or natural disasters	√	√		√	√	√
Factors relating to resources (including human resource, funding, and materials)		√	√	√	√	√
Data were used to construct the studied strategies	√	√	√	√	√	√
**Policy objectives**						
To improve diet (e.g., quantity and quality)	√	√		√	√	√
To improve the nutrition status of mothers and children (including focus on protein energy malnutrition)	√	√	√	√	√	√
To improve micronutrient status (e.g., Iron, Zinc, Vitamin A, Vitamin D)		√			√	√
To prevent and control overweight, obesity, or other chronic diseases		√			√	√
To prevent and control of infectious disease (including foodborne and waterborne diseases)						
To improve knowledge and practices regarding nutrition in the general population	√	√		√	√	√
To strengthen the national or local health system, or to reinforce the capacity and effectiveness of the nutrition services network in both community and health care facilities		√	√	√	√	√
To reduce inequities or barriers in access to care or to reduce nutrition and health disparities	√	√	√			√
