# Supplementary material for: Bridging the evidence-to-action gap: enhancing alignment of national nutrition strategies in Cambodia, Laos, and Vietnam with global and regional recommendations
Source: Front Nutr. 2024 Jan 8;10:1277804. doi: 10.3389/fnut.2023.1277804 (PMC10800738; doi:10.3389/fnut.2023.1277804)
Supplement: Supplementary file 2 [file Data_Sheet_2.pdf]

Appendix 2. Specific roles of stakeholders involved in national nutrition strategies, by country

	Cambodia		Laos		Vietnam	
	2014-2018	2019-2023	2016-2020	2021-2025	2011-2020	2021-2030
**Funding sources**						
National level	√	√	√	√	√	√
Sub-national levels		√		√	√	√
Civil society organizations, or unions[a]		√		√		
International organizations, or donors[b]		√		√	√	
Private sector				√		
**Technical support**						
National level	√	√	√	√		√
Sub-national levels	√	√	√	√		
Civil society organizations, or unions		√	√	√	√	√
International organizations, or donors	√	√	√	√		√
Private sector	√					√
Academic or research institutions	√			√	√	
**Implementation**						
National level	√	√	√	√	√	√
Sub-national levels	√	√	√	√	√	√
Civil society organizations, or unions	√	√	√	√	√	√
International organizations, or donors		√		√		
Private sector		√				

[a]Civil society organizations and unions include unions (Trade, Women, Farmers, and Youth), societies (Veterans, Teachers, Elderly), and religious, villages and tribe leaders. [b]International Organizations, donors include UNICEF, WHO, FAO, World Bank, other development bank (e.g., ADB), governments of other countries (e.g., USAIDS, Australian Aid, UK Aid), Foundations (e.g., Bill & Melinda Gates Foundation), research foundation, and international non-Governmental Organizations (NGOs), and In-country donors.
